# Back from the brink of obscurity

**DOI:** 10.7554/eLife.36649

**Published:** 2018-04-18

**Authors:** Donald C Vinh

**Affiliations:** 1 Department of MedicineMcGill University Health CentreMontrealCanada; 2 Department of Medical MicrobiologyMcGill University Health CentreMontrealCanada; 3 Department of Human GeneticsMcGill University Health CentreMontrealCanada; 4 Infectious Disease Susceptibility ProgramThe Research Institute of the McGill University Health CentreMontrealCanada

**Keywords:** Whipple's disease, primary immunodeficiency, IRF4, haploinsufficiency, Human

## Abstract

A mutation in a transcription factor makes people susceptible to *Tropheryma whipplei*, the bacterium that causes a rare condition called Whipple's disease.

**Related research article** Guérin A, Kerner G, Marr N, Markle JG, Fenollar F, Wong N, Boughorbel S, Avery DT, Ma CS, Bougarn S, Bouaziz M, Beziat V, Della Mina E, Oleaga-Quintas C, Lazarovt T, Worley L, Nguyen T, Patin E, Deswarte C, Martinez-Barricarte R, Boucherit S, Ayral X, Edouard S, Boisson-Dupuis S, Rattina V, Bigio B, Vogt G, Geissmann F, Quintana-Murci L, Chaussabel D, Tangye SG, Raoult D, Abel L, Bustamante J, Casanova JL. 2018. IRF4 haploinsufficiency in a family with Whipple's disease. *eLife* **7**:e32340. doi: 10.7554/eLife.32340

Throughout history, human health has been perpetually challenged by pestilence. In the late 19th century, 'germ theory' established the role of specific microbes in infectious diseases. However, it later became apparent that only a few of the individuals who are exposed to a microbe go on to develop a disease, and even fewer die. Why does this happen? Now, in eLife, Jean-Laurent Casanova and colleagues – including Antoine Guérin as first author – report one answer to this question for an obscure infection known as Whipple's disease (Guérin et al., 2018).

In 1907, George Whipple described a fatal disease that was marked by pain spreading through the joints, chronic diarrhea, excessive fat in the feces, and weight loss (Whipple, 1907). Under the microscope, he saw fatty material accumulate in the intestine and surrounding lymph nodes (Figure 1); he also noted abnormal 'foamy' cells with an associated rod-shaped microbe. Subsequent reports highlighted that this disease spread systemically through the body, affecting various organs.

**Figure 1.**
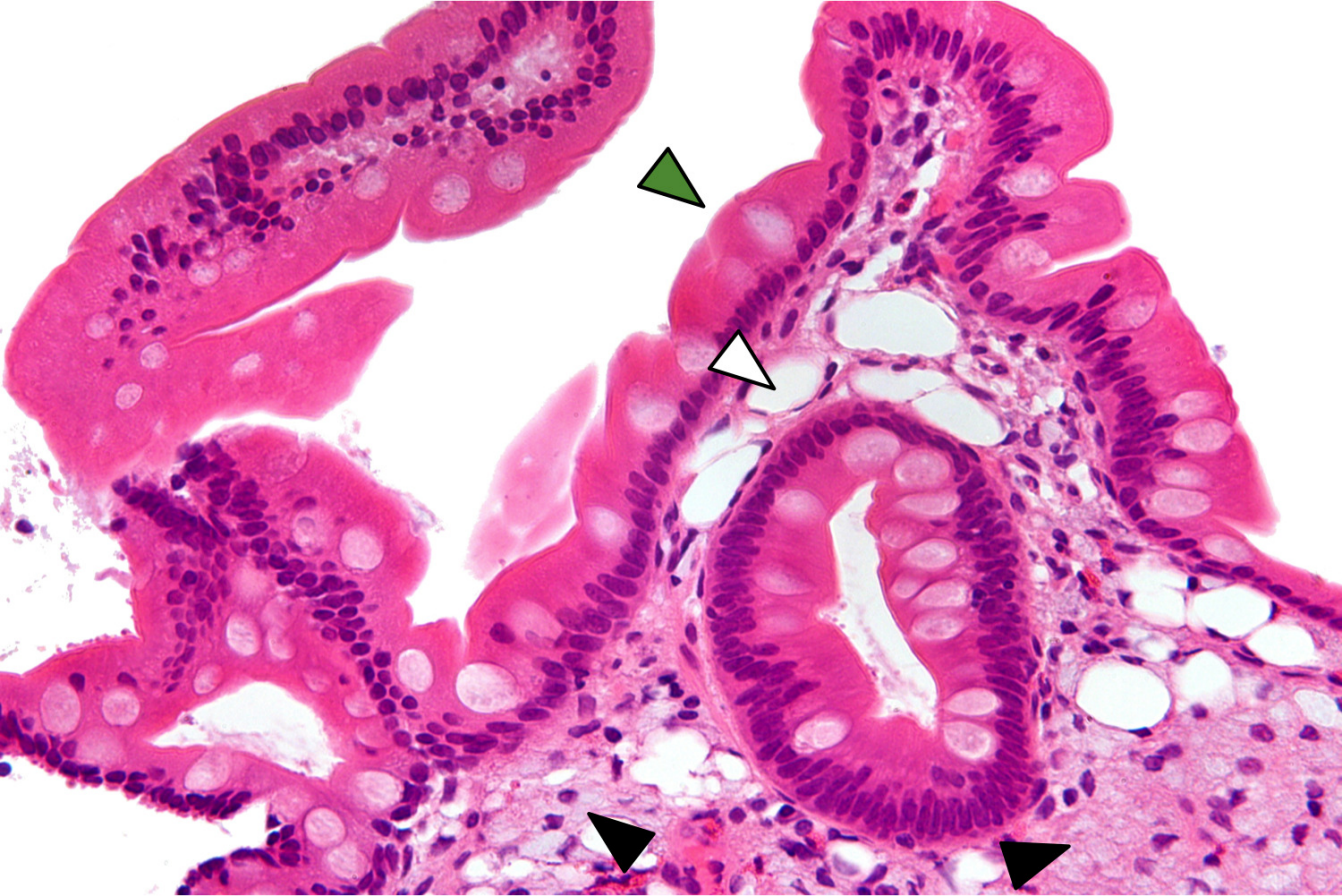
Biopsy of the small intestine showing features classic for Whipple’s disease. The epithelial cells that line the gut are smooth because the microvilli normally found on them have been blunted (green arrowhead). The area beneath the epithelial cells, the lamina propria, is filled with numerous round cells with a 'foamy' appearance: these are the macrophages (black arrowheads). Extracellular fat has also accumulated in this area (white arrowhead). The tissue is stained with hematoxylin and eosin stain.

IMAGE CREDIT: Micrograph by Nephron (CC BY-SA 3.0).

The bacterium was fastidious and could not be grown in the laboratory. In 1949, however, a new staining technique allowed the foamy cells, which are actually macrophages, to be recognized in affected tissues, thus enabling diagnosis of the disease (Black-Schaffer, 1949). Later, in 1961, electron microscopy identified the distinctive three layers of 'Whipple’s bacillus' (Yardley and Hendrix, 1961), and in 1992 genetic sequencing revealed it to be a previously unknown organism, which was designated *Tropheryma whippelii* and later renamed *Tropheryma whipplei* (Relman et al., 1992; La Scola et al., 2001). During this time it was also shown that antibiotics could transform Whipple’s disease into a treatable condition.

As with any disease, doctors seek to better understand the condition in order to improve diagnosis and treatment. PCR-based methods demonstrated that the organism was ubiquitous; for example, it was found in sewage water, human saliva and feces from people unaffected by Whipple’s disease (Maiwald et al., 1998; Street et al., 1999). Blood tests also revealed that human exposure was common (Raoult et al., 2000). So, why was the incidence of disease so low? Since *T. whipplei *could not be cultured conventionally, researchers could not develop experimental infection models to understand how the disease develops and what makes hosts susceptible. Thus, most researchers studied naturally occurring infections in humans, and made observations that were consistent with Whipple’s original description, including dysfunctional macrophages in affected patients.

Meanwhile, astute clinical observation opened an alternate line of investigations. In 1955, a familial case of Whipple’s disease was reported, involving a mother and only two of her five adult children. This implied a hereditary component of susceptibility (Puite and Tesluk, 1955), and while no further genetic studies were reported from that family, several similar cases were described in other families (Gross et al., 1959). These discoveries implied that genetic approaches could circumvent the microbe’s fastidiousness and finally provide insight into this obscure disease.

Guérin et al. – who are based in institutions in France, Qatar, the United States, and Australia – evaluated 26 members of a French family: four people had Whipple’s disease; five were carriers of the bacterium but did not have the disease; 13 were healthy non-carriers; and four were healthy individuals whose carrier status was unknown. Elegant genomic studies and bioinformatics analyses identified a new variant of *IRF4* (the gene for an immune transcription factor) in all those with the disease; in all the carriers; in two of the healthy non-carriers; and in one whose carrier status was unknown. The new variant was very rare and did not appear in any genomics database. Each individual carried one copy of the rare variant alongside a more typical version of the gene (i.e. they were all heterozygous).

Molecular investigations revealed that the rare variant differed by one amino acid (a tryptophan in place of an arginine). This change compromised the protein’s ability to bind to DNA and activate transcription. However, the mutant version of the protein did not interfere with the normal version, meaning it did not exert a negative dominance effect. Instead, Guérin et al. presumed the susceptibility occurs because people must need working protein from both versions of this gene to be protected against the bacteria. This phenomenon is referred to as haploinsufficiency.

In experiments that capitalized on the unique expertise of Didier Raoult's lab in culturing *T. whipplei* in vitro, the loss-of-function in IRF4 was linked to a distinct gene expression profile in response to the bacterium. Similar results were seen for a related bacterium, *Mycobacterium bovis* strain BCG. The genes that were expressed differently in response to *T. whipplei* could not be organized into a defined pathway, but those that differed in the response to BCG highlighted the role of IRF4 in regulating macrophages in cell-mediated immunity.

Together these findings raise a number of exciting questions. First, which IRF4-expressing cell types mitigate the disease? What molecular pathway connects IRF4 to the recognition and effective control or eradication of *T. whipplei*? What is the basis for the macrophages and lipid accumulation phenotypes that characterize this disease? And are mutations in IRF4 or other molecules at play in other patients with Whipple’s disease? These and other questions can now be pursued, guided by the study of this single yet informative family. On a grander scale, this work also reinforces the value of studying humans, including those with rare inborn errors of immunity, to understand human immunobiology, using approaches that cross disciplines.

## References

[bib1] Black-Schaffer B (1949). The tinctoral demonstration of a glycoprotein in Whipple's disease. Experimental Biology and Medicine.

[bib2] Gross JB, Wollaeger EE, Sauer WG, Huizenga KA, Dahlin DC, Power MH (1959). Whipple's disease; report of four cases, including two in brothers, with observations on pathologic physiology, diagnosis, and treatment. Gastroenterology.

[bib3] Guérin A, Kerner G, Marr N, Markle JG, Fenollar F, Wong N, Boughorbel S, Avery DT, Ma CS, Bougarn S, Bouaziz M, Beziat V, Della Mina E, Oleaga-Quintas C, Lazarovt T, Worley L, Nguyen T, Patin E, Deswarte C, Martinez-Barricarte R, Boucherit S, Ayral X, Edouard S, Boisson-Dupuis S, Rattina V, Bigio B, Vogt G, Geissmann F, Quintana-Murci L, Chaussabel D, Tangye SG, Raoult D, Abel L, Bustamante J, Casanova JL (2018). IRF4 haploinsufficiency in a family with Whipple's disease. eLife.

[bib4] La Scola B, Fenollar F, Fournier PE, Altwegg M, Mallet MN, Raoult D (2001). Description of *Tropheryma whipplei* gen. nov., sp. nov., the Whipple's disease bacillus. International Journal of Systematic and Evolutionary Microbiology.

[bib5] Maiwald M, Schuhmacher F, Ditton HJ, von Herbay A (1998). Environmental occurrence of the Whipple's disease bacterium (*Tropheryma whippelii*). Applied and Environmental Microbiology.

[bib6] Puite RH, Tesluk H (1955). Whipple's disease. The American Journal of Medicine.

[bib7] Raoult D, Birg ML, La Scola B, Fournier PE, Enea M, Lepidi H, Roux V, Piette JC, Vandenesch F, Vital-Durand D, Marrie TJ (2000). Cultivation of the bacillus of Whipple's disease. New England Journal of Medicine.

[bib8] Relman DA, Schmidt TM, MacDermott RP, Falkow S (1992). Identification of the uncultured bacillus of Whipple's disease. New England Journal of Medicine.

[bib9] Street S, Donoghue HD, Neild GH (1999). *Tropheryma whippelii* DNA in saliva of healthy people. The Lancet.

[bib10] Whipple GH (1907). A hitherto undescribed disease characterized anatomically by deposits of fat and fatty acids in the intestinal and mesenteric lymphatic tissues. Bulletin of the Johns Hopkins Hospital.

[bib11] Yardley JH, Hendrix TR (1961). Combined electron and light microscopy in Whipple's disease. Demonstration of "bacillary bodies" in the intestine. Bulletin of the Johns Hopkins Hospital.

